# Implementation of outpatient hip and knee arthroplasty in a multicenter public healthcare setting

**DOI:** 10.2340/17453674.2024.40185

**Published:** 2024-05-07

**Authors:** Oddrún DANIELSEN, Claus VARNUM, Christian Bredgaard JENSEN, Thomas JAKOBSEN, Mikkel Rathsach ANDERSEN, Manuel Josef BIEDER, Søren OVERGAARD, Christoffer Calov JØRGENSEN, Henrik KEHLET, Kirill GROMOV, Martin LINDBERG-LARSEN

**Affiliations:** 1 Center for Fast-track Hip and Knee Replacement; 2 Department of Orthopaedic Surgery and Traumatology, Odense University Hospital and Svendborg; 3 Department of Orthopaedic Surgery, Lillebaelt Hospital – Vejle; 4 Department of Orthopaedic Surgery, Hvidovre University Hospital; 5 Department of Orthopaedic Surgery, Aalborg University Hospital; 6 Department of Orthopaedic Surgery, Copenhagen University Hospital, Herlev-Gentofte; 7 Department of Orthopaedic Surgery, Næstved, Slagelse and Ringsted Hospitals; 8 Department of Orthopaedic Surgery and Traumatology, Copenhagen University Hospital, Bispebjerg; 9 Department of Anaesthesia, Hospital of Northern Zealand, Hillerød; 10 Section of Surgical Pathophysiology, Copenhagen University Hospital, Rigshospitalet, Denmark

## Abstract

**Background and purpose:**

Length of hospital stay after hip and knee arthroplasty is about 1 day in Denmark with few patients discharged on the day of surgery. Hence, a protocol for multicenter implementation of discharge on day of surgery has been instituted. We aimed to describe the implementation of outpatient hip and knee arthroplasty in a multicenter public healthcare setting.

**Methods:**

We performed a prospective multicenter study from 7 public hospitals across Denmark. Patients were screened using well-defined in- and exclusion criteria and were discharged on day of surgery when fulfilling functional discharge criteria. The study period was from September 2022 to February 2023 with variable start of implementation. Data from the same centers in a 6-month period before the COVID pandemic from July 2019 to December 2019 was used for baseline control.

**Results:**

Of 2,756 primary hip and knee arthroplasties, 37% (95% confidence interval [CI] 35–39) were eligible (range 21–50% in centers) and 52% (range 24–62%) of these were discharged on day of surgery. 21% (CI 20–23) of all patients (eligible and non-eligible) were discharged on day of surgery with a range of 10–31% within centers. This was an additional 15% (CI 13–17, P < 0.001) compared with patients discharged in the control period (6% in 2019).

**Conclusion:**

We found it possible to perform outpatient hip and knee replacement in 21% of patients in a public healthcare setting, probably to be increased with further center experience.

There have been significant developments in hip and knee arthroplasty during the past decades with the introduction of fast-track protocols resulting in a reduction in postoperative length of hospital stay (LOS), postoperative morbidity, and costs [1-5]. Safe outpatient arthroplasty with discharge on day of surgery may be the ultimate goal of fast-track arthroplasty [6].

Previous single-center studies have shown the potential to discharge patients on the day of surgery after primary total hip arthroplasty (THA), total knee arthroplasty (TKA), and unicompartmental knee arthroplasty (UKA) [7-10]. Although selective use of outpatient surgery in individual centers has been shown to be feasible, the use of outpatient surgery on a national level is low [11,12]. Therefore, the study group designed a detailed protocol for the implementation of outpatient surgery in a multicenter collaboration across all regions in Denmark [13].

Hence, the aim of our study was to investigate the implementation of discharge on the day of surgery after primary hip and knee arthroplasty in a multicenter setting during the first 6-month study period.

## Methods

### Study design

The study was designed as a prospective multicenter cohort study. All reporting was performed according to the REporting of studies Conducted using Observational Routinely-collected Data (RECORD) guideline [14].

### Setting

“The Center for Fast-track Hip and Knee Replacement” is a multicenter collaboration consisting of 8 public arthroplasty centers across all 5 regions in Denmark, covering approximately 40% of the annual number of hip and knee arthroplasties in Denmark [9,15]. Only 3 of the 8 centers had previous experience with use of outpatient surgery before the study period. An educational program was introduced supporting that all centers followed the same protocol [13]. The study period was from September 1, 2022, to February 28, 2023, with centers labelled A–G. 1 center (Hospital Unit West, Gødstrup) did not participate because of major logistical changes due to transfer to a new hospital site and therefore did not include patients. Furthermore, 4 of the centers were only ready to start inclusion from October 1, 2022.

### Study population

The study population included patients receiving a primary elective THA, TKA, or UKA and included in the REDCap database at the 7 study centers in the implementation period. Patients were included after informed consent. The informed consent was in writing and necessary in order to gain full access to patient files and to send out questionnaires to the patients. Inclusion and exclusion criteria for planned outpatient surgery are presented in Table 1. Patients were discharged on day of surgery if fulfilling predefined discharge criteria (Table 2). The data completeness on center level is provided in Table 3 (see Appendix).

**Table 1:** Inclusion and exclusion criteria for planned discharge on day of surgery

**Inclusion criteria**
Unilateral elective primary THA, TKA, or UKA Age 18–80
**Exclusion criteria**
Acute myocardial infarction, cerebrovascular accident, transient ischemic attack, or coronary atherosclerotic disease within last 3 months Unstable ischemic heart disease Ejection fraction < 40% Glomerular filtration rate < 60 mL/min/1.73 m^2^ Chronic obstructive pulmonary disease with home oxygen Insulin-dependent diabetes mellitus Sleep apnea requiring mechanical treatment CFS ≥ 4^a^ 2 or more falls within last 3 months Body mass index < 18.5 or > 40 Not interested in discharge on day of surgery No adult present at home during the initial postoperative night^b^

a CFS = Clinical Frailty Scale [25].

b This criterion was inadvertently omitted from the protocol paper but has consistently been applied across all centers.

**Table 2:** Criteria for discharge on day of surgery

Activity level
Steady gait with crutches No dizziness during mobilization Can use stairs, if required by participant’s home environment
Postoperative nausea and vomiting
Minimal and efficiently treated with or without medication EWS^a^ < 2 Patients with EWS ≥ 2 must be discussed with a doctor prior to discharge
Pain
Numeral rating scale (NRS) (0–10, with 0 being no pain and 10 being the worst pain imaginable) NRS < 3 at rest NRS < 5 when walking 5 m or otherwise, acceptable level of pain assessed by the participant regardless of NRS score
Postoperative bleeding
Should be consistent with expected blood loss for this procedure and not require repeated dressing change

a EWS = national implemented Early Warning Score systems based on NEWS2 from the Royal College of Physicians [26].

**Table 3:** Data completeness on center level

Number of surgeries, n (%)	Sept.	Oct.	2022		2023		
			Nov.	Dec.	Jan.	Feb.	Total
Hospital A							
according to department	–	26	54	40	70	41	231
registered in REDCap	–	7 (27)	22 (41)	20 (50)	38 (54)	21 (51)	108 (47)
Hospital:B							
according to department	57	43	67	50	89	58	364
registered in REDCap	21 (37)	39 (91)	63 (94)	43 (86)	78 (88)	56 (97)	300 (82)
Hospital C							
according to department	114	161	160	114	192	147	888
registered in REDCap	71 (62)	140 (87)	149 (93)	100 (88)	183 (95)	135 (92)	778 (88)
Hospital D							
according to department	–	81	100	55	83	64	393
registered in REDCap	–	42 (52)	83 (83)	43 (78)	75 (90)	59 (92)	302 (77)
Hospital E							
according to department	60	150	174	129	168	116	797
registered in REDCap	15 (25)	53 (35)	70 (40)	80 (62)	114 (68)	86 (74)	418 (52)
Hospital F							
according to department	70	60	71	48	68	62	379
registered in REDCap	69 (99)	56 (93)	65 (92)	43 (90)	63 (93)	61 (98)	357 (94)
Hospital G							
according to department	–	102	167	116	176	127	687
registered in REDCap	–	8 (7.8)	102 (61)	110 (95)	160 (91)	113 (89)	493 (72)

**Figure 1:**
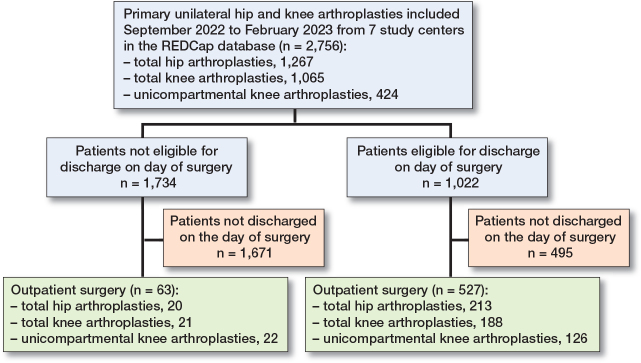
Flowchart.

Patients eligible for outpatient surgery were scheduled with intended start of surgery before 1 pm. (Figure 1).

A historic control cohort with data on the number of outpatient procedures from the study centers in a 6-month period from July 1, 2019, to December 31, 2019 was used for comparison.

### Deviation from study protocol

The focus of our study was to document and describe the implementation process following the detailed protocol designed by the study group prior to the implementation process.

We occasionally noticed a discrepancy in the exclusion criteria published in the protocol paper and those used in practice. The exclusion criterion “no adult present at home during the initial postoperative night” is not mentioned in the published protocol, and our study therefore deviates from study protocol regarding this criterion.

### Data sources

Data was prospectively collected by dedicated research staff at the individual study sites with physician back-up if necessary and stored online in a REDCap database in collaboration with the Open Patient data Explorative Network (OPEN) in Odense University Hospital. The database includes patient-reported data as well as detailed data on patient comorbidities and medications collected prospectively by research staff [13].

Data on the historical baseline control group was obtained from the Danish National Patient Registry (DNPR), which contains specific procedure codes, diagnosis codes as well and discharge dates from all hospitalizations since 1977 [15].

### Statistics

A preliminary power calculation was performed to ensure the feasibility of the study within the planned study period. To detect a 10-percentage point increase in discharge on the day of surgery, we required a sample size of 266 patients in each group, ensuring a power of 90% and an alpha level of 5%, and this was fully achieved with a 6-month study period [13].

A before-and-after design was used, and outcomes presented as proportions with 95% confidence intervals (CI). An estimate of the intervention effect was quantified through the measurement of the difference in proportions using Stata Statistical Software: Release 18 (StataCorp LLC, College Station, TX, USA).

### Ethics, registration, funding, and disclosures

The study was preregistered in the Region of Southern Denmark and approval for data storage and management of study-associated data was obtained. The study was also registered on ClinicalTrials.gov (NCT05613439).

Treatment of eligible patients for outpatient surgery was standard of care at the participating centers according to the described protocol [13] and ethical approval was therefore not required.

“The Center for Fast-track Hip and Knee Replacement” collaboration [15] was funded in 2021 from the NOVO Nordisk Foundation (Grant number: NNF21SA0073760) to support the overall fast-track project, including research staff at all centers, data management and follow-up on complications. Furthermore, salary for the PhD student (OD) was funded from the Candy’s Foundation, University of Southern Denmark and Region of Southern Denmark. HK and KG are members of the Zimmer Biomet advisory board on rapid recovery, and CV received travel expenses from Stryker with no relevance to the present study. The remaining authors report no conflicts of interest related to this project. Completed disclosure forms for this article following the ICMJE template are available on the article page, doi: 10.2340/17453674.2024.40185

## Results

The study cohort included 2,756 patients with 1,267 primary THAs, 1,065 primary TKAs, and 424 primary UKAs. The mean age was 69 years and 58% were females (Table 4).

**Table 4:** Patient demographics. Values are percentages unless otherwise specified

Factor	Entire study cohort	Eligible patients n = 1,022		Non-eligible patients n = 1,734	
		DOS	Not DOS	DOS	Not DOS
Surgical procedure, n	2,756	527	495	63	1,671
THA	1,267	213	223	20	846
TKA	1,065	188	184	21	615
UKA	424	126	88	22	210
Mean age, years	69	65	66	67	71
Sex					
Female	58	48	55	59	61
Male	42	52	45	41	39
Mean body mass index	29	27	29	28	30
Cohabitation					
Cohabiting	73	91	87	69	62
Living alone	27	9	13	31	38
Mean CFS	2.5	2.1	2.2	2.6	2.8
Pharmacologically treated					
diabetes mellitus	9	5	8	6	11
heart disease	58	48	51	51	64
pulmonary disease	26	11	12	13	34

DOS = discharged on the day of surgery.

CFS = Clinical Frailty Scale [25].

**Figure 2:**
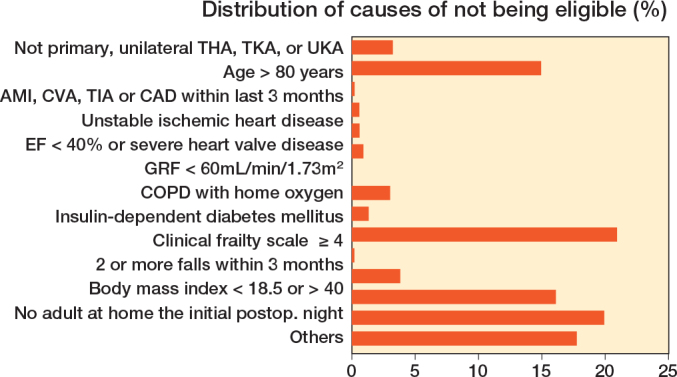
Causes of not being eligible for discharge on day of surgery. AMI = acute myocardial infarction. CVA = cerebrovascular accident. TIA = transient ischemic attack. CAD = coronary atherosclerotic disease. EF = ejection fraction. GFR = glomerular filtration rate. COPD = chronic obstructive pulmonary disease. CFS = Clinical frailty scale. Others = e.g., cognitively impaired patient, elderly/ill cohabitant, requiring ferry transport to get home, etc.

**Figure 3:**
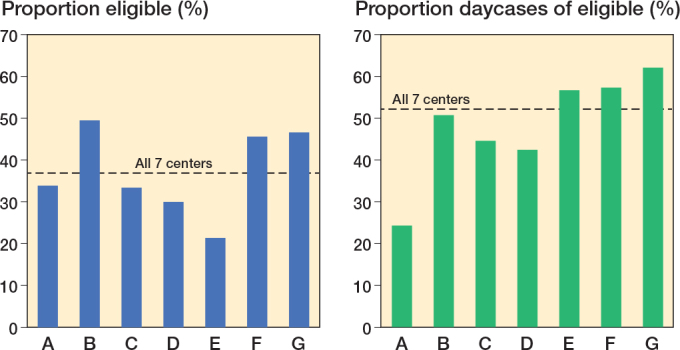
(Left panel) Patients found eligible for discharge on day of surgery on center level (A–G). (Right panel) Proportion of eligible patients discharged on the day of surgery on center level (A–G).

1,022 patients (37%, CI 35–39) were found eligible for discharge on day of surgery (Figure 1). The proportion of patients eligible ranged from 21% to 50% between centers and causes of not being eligible are presented in Figure 2. 52% (CI 49–55) of the eligible patients were discharged on the day of surgery, with a range of 24–62% between the centers (Figure 3). 92% of the eligible patients had start of surgery before 1 pm as intended according to the study protocol.

**Figure 4:**
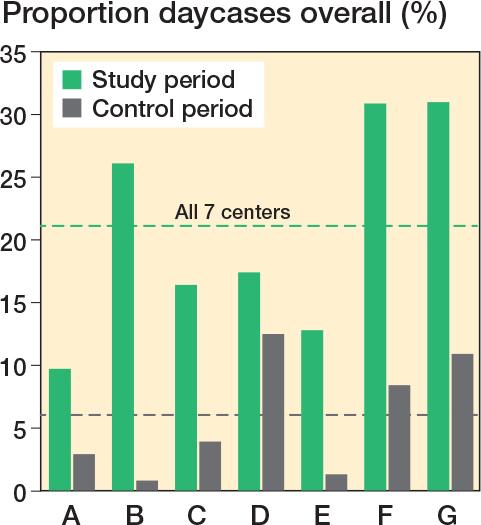
Overall discharge on day of surgery on center level (A–G) in the study period (September 1, 2022–February 28, 2023) versus the control period (July 1, 2019–December 31, 2019).

21% (CI 20–23) of all patients (eligible and non-eligible) were discharged on the day of surgery (range 10–31% between centers) (Figure 4). There was a statistically significant difference in the overall proportion of patients discharged on day of surgery between 2019 (control period 6%) and 2023 (study period 21%), with a difference of 15% (CI 13–17, P < 0.001). 36% of UKAs, 20% of TKAs, and 18% of THAs were discharged on day of surgery.

The distribution of patients discharged on the day of surgery was relatively constant during the 6-month implementation period.

## Discussion

We aimed to describe the implementation of outpatient hip and knee arthroplasty in a multicenter public healthcare setting, and we found an increase in outpatient surgery from 6% at baseline to 21% during the implementation period of the study protocol. To our knowledge, this is the first multicenter study with a study protocol including well-defined eligibility criteria and discharge criteria from a public healthcare setting reporting the feasibility of multicenter implementation of outpatient hip and knee replacement.

Despite an increasing interest in outpatient surgery worldwide, the eligibility criteria are still debatable. In- and exclusion criteria in our study were based on previous data from outpatient surgery [16-17] as well as our previous multicenter data on the risk of complications after fast-track hip and knee arthroplasty [4].

52% of the eligible patients were discharged on the day of surgery, where previous prospective studies have reported huge variations in success from 24–99% [7-10,17-19]. There are several possible explanations for these variable results. First, the selection of patients in the studies varied and was not always based on well-defined in- and exclusion criteria [9,18,20-21]. Furthermore, some studies were performed in ambulatory day care center settings with very restrictive eligibility criteria including mainly healthy young patients [8,10]. Finally, previous study cohorts were rather small (n ≤ 200 patients) making generalizability and interpretation difficult [8-10,18].

In our population representing participants from all regions of the country, the frequency of patients found eligible for discharge on the day of surgery varied considerably between the centers. 5 of the centers receive mainly elective patients whereas 2 centers have both acute and elective patients on the same site. Furthermore, the individual surgeon’s interpretation of the protocol and the concept of discharge on day of surgery may play a role despite the pre-study educational program being instituted at all centers [13].

We also found important differences in the proportion of patients discharged on day of surgery among centers. Only 3 of the study centers (center D, F, G in Figure 4) had previous experience with discharge on day of surgery, and 2 of these centers improved considerably from about 10% at baseline to about 30% in the study period. 4 centers had no pre-study experience with discharge on day of surgery and 3 centers reached 10% of outpatient surgery with 1 center reaching 26%. Surprisingly, we did not observe any significant increase in the proportion discharged on day of surgery during the 6-month study period, requiring further study over a longer time period. Nevertheless, based on this initial data, we estimate it to be realistic to achieve an overall 30% discharge on day of surgery in our multicenter collaboration within the following years.

The overall frequency of patients discharged on day of surgery reached 21% in the implementation period of our study. In comparison, Coenders et al. reached 34% of 257 patients discharged on the day of surgery in their prospective study over 3.5 years. The proportion of patients meeting the inclusion criteria (40%) was at the same level as our study (37%), but they included only primary THAs from a single private center [7].

The strength of our study was the prospective design with a well-defined setup [13]. Furthermore, our study included data from a multicenter collaboration contributing about 40% of all annual primary hip and knee arthroplasties in Denmark [22,23] from all Danish regions and from a socialized healthcare setting, which may increase the generalizability of the results. The multicenter collaboration was originally established in 2009, and thus was already well-functioning before the implementation of the protocol for this study [15].

We acknowledge that time trends throughout the study period may have introduced confounding factors in our before-and-after analysis. Additionally, diverse logistical barriers at individual centers could have influenced our results, despite adherence to the same protocol, which included a pre-study educational program [13]. Further investigation of the center type aspect is warranted and a subject for upcoming studies. Another limitation of the study was that not all centers reached acceptable completeness of data in the REDCap database during the implementation period (Table 3, see Appendix). Reasons for incompleteness were primarily related to logistical challenges with implementation of the database on center level. A few patients also refused to provide informed consent to registration within the REDCap database, which might introduce selection bias.

The internal logistic problems have been solved with all centers having reached 90% data completeness after the implementation period. However, we also acknowledge that the overall frequency of patients discharged on day of surgery may be overestimated, as we have no information on patients not included in the database. Finally, our collaboration focuses not only on the speed of discharge, but also on the safety of the outpatient setup [24]. This important aspect will be secured in future studies by complete follow-up through the established obligatory health registers in Denmark. Nevertheless, our current study aimed to describe the feasibility of implementation of our study protocol for outpatient surgery and may provide valuable information for setting realistic goals when planning an outpatient protocol in other institutions.

### Conclusion

During the implementation period we found it possible to perform outpatient hip and knee arthroplasty in 21% of patients in a multicenter public healthcare setting.

## Notes

CV, TJ, MRA, MJB, SO, CCJ, HK, KG, and MLL are members of the Center for Fast-track Hip and Knee Replacement steering committee. The study protocol and guideline were planned at steering committee meetings. OD and CBJ undertook data gathering. OD and MLL drafted the manuscript. All authors reviewed the manuscript.

Handling co-editors: Bart Swierstra and Philippe Wagner

*Acta* thanks Nina Mathijssen for help with peer review of this manuscript.
